# Supplementary material for: Ultrafast photothermoelectric effect in Dirac semimetallic Cd3As2 revealed by terahertz emission
Source: Nat Commun. 2022 Mar 25;13:1623. doi: 10.1038/s41467-022-29168-w (PMC8956572; doi:10.1038/s41467-022-29168-w)
Supplement: Supplementary file 1 — Supplementary Information [file 41467_2022_29168_MOESM1_ESM.pdf]

Supplementary Information

Ultrafast photothermoelectric effect in Dirac semimetallic Cd_3As_2 revealed by terahertz emission

Wei Lu[1,2], Zipu Fan[2], Yunkun Yang[3], Junchao Ma[2], Jiawei Lai[2], Xiaoming Song[1], Xiao Zhuo[2], Zhaoran Xu[2], Jing Liu[1], Xiaodong Hu[1], Shuyun Zhou[4,5], Faxian Xiu[3], Jinluo Cheng[6] & Dong Sun[2,5,*]

[1]State Key Laboratory of Precision Measurement Technology and Instruments, School of Precision Instruments and Opto-electronics Engineering, Tianjin University, Tianjin 300072, China

[2]International Center for Quantum Materials, School of Physics, Peking University, Beijing 100871, China

[3]State Key Laboratory of Surface Physics and Department of Physics, Fudan University, Shanghai 200433, China

[4]State Key Laboratory of Low Dimensional Quantum Physics and Department of Physics, Tsinghua University, Beijing, 100084, China

[5]Collaborative Innovation Center of Quantum Matter, Beijing 100871, China

[6]Changchun Institute of Optics, Fine Mechanics and Physics, Chinese Academy of Sciences, Changchun 130033, China

[*]Corresponding author. Email: sundong@pku.edu.cn (D.S.)

Supplementary Note 1. Thermoelectric coefficients of different materials

Supplementary Table 1 summarize the Seebeck (S_{xx}) and Nernst (S_{xy}) coefficients of typical topological semimetals, conventional metallic and semiconducting thermoelectric materials, and materials with Nernst coefficients that are comparable with Seebeck coefficients, which are usually called “giant Nernst effect materials”. We note many topological semimetals are excellent thermoelectric materials and thermoelectricity serves as one of their most important potential applications.

Table 1. Seebeck (S_{xx}) and Nernst (S_{xy}) coefficients of typical topological semimetals, conventional thermoelectric materials, and giant Nernst effect materials.

Materials	S_{xx} (μV/K)	S_{xy} (μV/K)
Cd_3As_2 [a]	~200 (150 K, 0 T)[1]	~160 (200 K, 10 T)[2]
$ZrTe_5$ [a]	~500 (100 K, 13T)[3]	~5000 (100 K, 13 T)[3]
NbAs [a]	~450 (100 K, 14 T)[4]	~500 (140 K, 14 T)[4]
NbP [a]	~1100 (30 K, 12 T)[4]	~800 (109 K, 9 T)[5]
TaAs [a]	~750 (35 K, 10 T)[4]	~1800 (60 K, 14 T)[4]
TaP [a]	~1700 (150 K, 0 T)[4]	~1700 (150 K, 0 T)[4]

WTe_2 [a]	~90 (300 K, 0 T)[6]	~4000 (3.7 K, 17 T)[7]
PbS [b]	~400 (300 K, 0 T)[8]	NA
SnSe [b]	~550 (300 K, 0 T)[9]	NA
Mg_2Si [b]	~150 (300 K, 0 T)[10]	NA
Au [b]	~9 (5 K, 0 T)[11]	~10^{-4} (μV/K T)[12]
$YbAl_3$ [b]	~80 (300 K, 0 T)[13]	~0.22 (30 K, 2 T)[13]
$SrTiO_3$ [b]	~180 (6 K, 0 T)[14]	~9 (0.9 K, 10 T)[14]
PbTe [b]	265~510 (300 K, 0 T)[15]	>25 (100 K, 1.5 T)[15]
URu_2Si_2 [c]	~22 (15 K, 0 T)[12]	~30 (5 K, 12 T)[12]
$BaFe_2As_2$ [c]	~8 (170 K, 0 T)[16]	~29 (45 K, 30 T)[16]
$P_4W_{12}O_{44}$ [c]	~70 (15 K, 0 T)[17]	~13 (15 K, 9 T)[17]

[a] topological semimetals
[b] conventional thermoelectric materials
[c] giant Nernst effect materials

Supplementary Note 2. THz detection by electro-optical sampling with ZnTe crystal

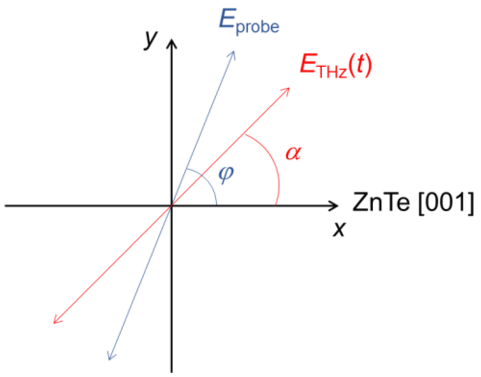

Supplementary Fig. 1 E-O sampling geometry. Configuration of the THz electric field (E_{THz}) direction and 800-nm sampling beam polarization (E_{probe}) direction with respect to the ZnTe [001] axis.

The electro-optical (E-O) sampling signal $S(t, \alpha, \varphi)$ of linearly polarized THz wave detected by standard E-O sampling method using ZnTe (110) crystal can be expressed as[18]:

$$S(t, \alpha, \varphi) \propto E_{THz}(t)\ (2\ \sin\alpha\ \cos2\varphi + \cos\alpha\ \sin2\varphi) \quad (1)$$

where α and φ are the polarization angles of the THz wave and the 800-nm sampling beam with respect to the [001] direction of ZnTe crystal as shown in Supplementary Fig. 1, t is the time delay of the sampling beam, and $E_{THz}(t)$ is the electric field of THz wave. If the ZnTe [001] axis is along x-axis, and the polarization of sampling beam E_{probe} is along y-axis ($\varphi = \pi/2$), Supplementary Eq. 1 becomes:

$$S(t, \alpha) \propto 2\ E_{THz}(t)\ \sin(\alpha + \pi) = -2\ E_{THz}(t)\ \sin\alpha \quad (2)$$

Therefore, the ZnTe crystal is acting like a polarizer and the detected THz signal follows a sinusoidal

function of the THz polarization angle. When E_{THz} is along the ZnTe [001] axis, $\alpha = 0$, $S(t, 0) = 0$, which corresponds to no E-O sampling signal. Alternatively, we can align the ZnTe [001] axis along y-axis (φ = 0) so that x-polarized component of THz wave are maximized in this E-O sampling geometry ($S(t, \alpha) \propto -2\, E_{\mathrm{THz}} \cos\alpha$).

If the THz wave is elliptically polarized, $\boldsymbol{E}(t) = e^{-i(\omega t+\alpha_0)}(E_x, iE_y)$, where α_0 is the initial phase of the elliptical light, which depends on the azimuthal angle (θ) of the sample. Therefore, according to the Supplementary Eq. 2, the peak position of the THz waveform will have a shift when varying θ.

Supplementary Note 3. Detection of THz emission from in-plane and out-of-plane transient currents

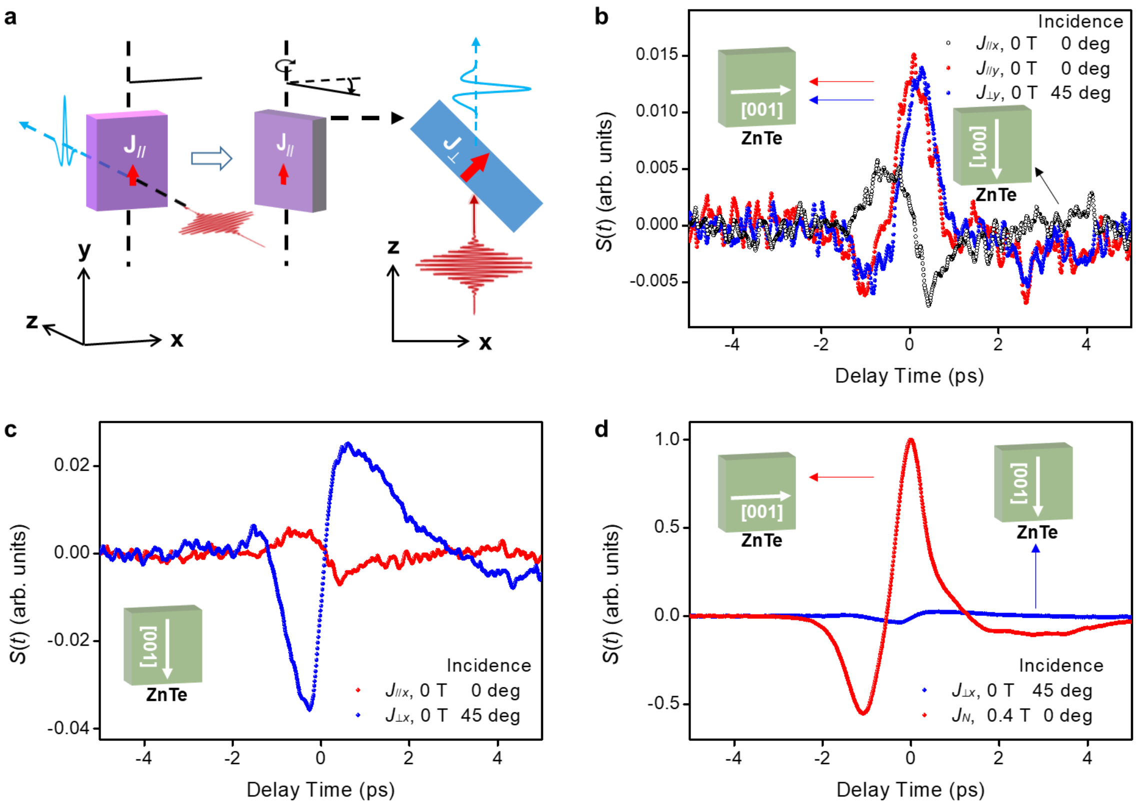

Supplementary Fig. 2 THz emission from in-plane and out-of-plane transient currents. a Side view of normal incidence and oblique incidence geometry of excitation beam (left two configurations) and top view of oblique incidence (right configuration). **b** THz emission for normal incidence and 45°-oblique incidence geometry excitation. In the detection geometry, the [001] direction of ZnTe is set on x-axis, so the electric field component of THz emission along y-axis is detected. **c** Then the [001] direction of ZnTe is set on y-axis, so the electric field component of THz emission along x-polarization is detected. **d** THz waveform at 0.4 T for normal incidence with the [001] direction of ZnTe set on x-axis and THz waveform for 45°-incidence excitation at 0 T with the [001] direction of ZnTe set on y-axis. The amplitude of THz signals at 0.4 T is set as 1 in the plot.

As shown in Supplementary Fig. 2a, under normal incidence, only the THz wave emitted by the in-

plane transient current ($J_{//}$) can propagate along the light path (along z-axis), thus can be detected according to the experimental geometry. The propagation of THz wave emitted by the out-of-plane transient current ($J_{\perp}$) is perpendicular to z-axis under normal incidence, so it cannot be detected in the E-O sampling configuration. When the sample is excited with an oblique angle, $J_{\perp}$ can have a nonzero projection along x-direction ($J_{\perp x}$) and the THz emission from $J_{\perp x}$ can be detected. To distinguish the THz emission contribution from $J_{\perp x}$ under the oblique excitation condition, the x-component of the in-plane current $J_{//}$ need to be minimized to avoid overlapping with THz emission signal from $J_{\perp x}$.

i. In-plane current detection

First, we set the ZnTe [001] along x-axis to detect the y-polarized THz wave according to the discussion in Supplementary Note 2. The azimuthal angle of sample is varied to obtain optimum THz signal, so that $J_{//}$ is along y-axis to get optimized THz detection efficiency. Then we fixed the ZnTe [001] along y-axis to detect the x-polarized THz wave. At normal incidence, the x-component of $J_{//}$ is significantly smaller than the y-polarized component as shown in Supplementary Fig. 2b ($J_{//y}/J_{//x}$ ~2.9), here $J_{//x}$ survives because imperfect optimization of $J_{//y}$ due to the limited THz signal when rotating the azimuthal angle of sample. When the sample is rotated along y-axis by 45° (middle configuration of Supplementary Fig. 2a) to have 45°-oblique incident excitation, the THz waveform obtained with ZnTe [001] along x-axis almost keeps the same as shown in Supplementary Fig. 2b, because $J_{//y}$ contributes to the detected THz signal in both configurations.

ii. Out-of-plane current detection by oblique incidence

The out-of-plane current $J_{\perp}$ is only detectable when we set the ZnTe [001] along y-axis. At 45°-oblique incidence, $J_{\perp}$ has a component projected onto x-axis （$J_{\perp x}$）, which provides detectable THz signal as shown in Supplementary Fig. 2c. The peak-to-peak amplitude of $J_{\perp x}$ is about 3 times larger than $J_{//y}$, implying $J_{\perp}$ is much stronger than $J_{//}$.

iii. Out-of-plane current detection by applying an in-plane magnetic field

Alternatively, instead of rotating the sample along y-axis to have an oblique incidence, an in-plane magnetic field can be applied to deflect the direction of $J_{\perp}$ to provide a detectable transient current (J_N) in a classical picture, which corresponds to Nernst response in this work. Experimentally, by applying an in-plane magnetic field of 0.4 T, the emitted THz signal is one order of magnitude larger than that for 45°-oblique incidence at 0 T as shown in Supplementary Fig. 2d and maintext Fig. 1d.

iv. Comparison of THz spectra

The THz waveforms generated from $J_{//y}$ at normal incidence, $J_{\perp x}$ under 45°-oblique incidence and J_N under normal incidence with 0.4 T magnetic field and their Fourier transform spectra are shown in Supplementary Fig. 3a and 3b respectively. At normal incidence, the THz waveforms for B = 0 T and 0.4 T are similar after normalization as shown in Supplementary Fig. 3a; while at 45°-oblique incidence, the THz waveform is very different, suggesting the transport dynamics may be different for in-plane and out-of-plane transient current. In the frequency domain (Supplementary Fig. 3b), the central frequency of emitted THz spectrum (0.18~0.25 THz) is slightly different for B = 0 T and 0.4 T at normal

incidence and the peak of 0.4 T has a flat and broader top. The full widths at half maximum (FWHM) of the three THz pulses are all at about 0.5 THz.

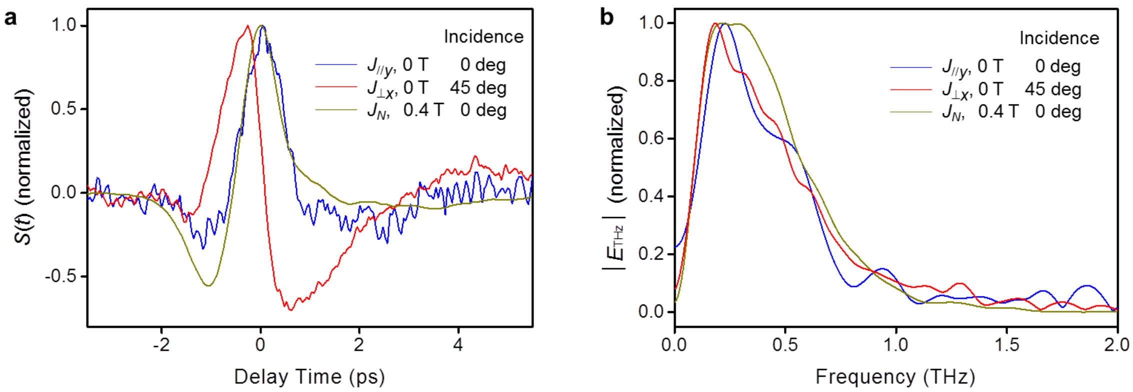

Supplementary Fig. 3 THz spectra. a The normalized THz waveforms obtained at a magnetic field intensity of 0 T and 0.4 T respectively. **b** The normalized Fourier spectra.

Supplementary Note 4. Excitation power dependence of THz emission at *B* = 0 T

Under different excitation intensity, the THz waveforms are very similar, but the maximum amplitude has a shift of ~0.3 ps as the laser intensity increases from 12.5 mW to 225 mW as shown in Supplementary Fig. 4b. As a consequence, the frequency spectrum has a blue shift (Supplementary Fig. 4c and 4d), indicating a faster establishment of the transient current when excitation power increases.

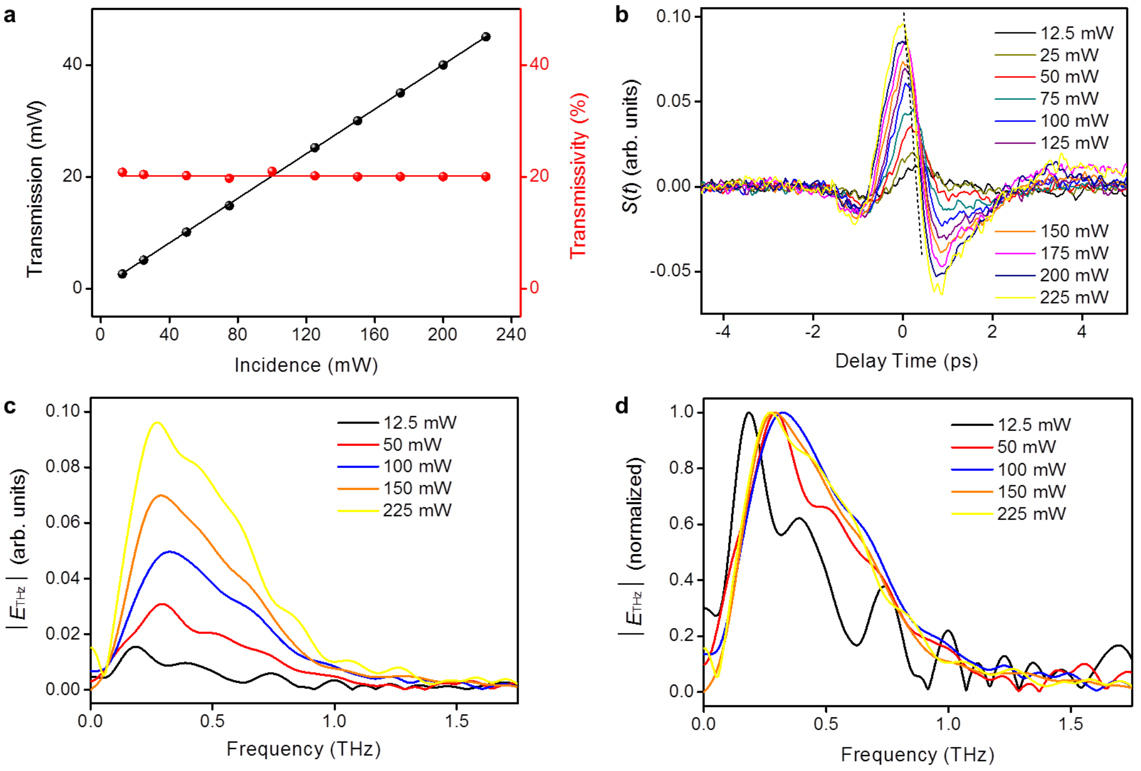

Supplementary Fig. 4 Excitation power dependent measurements of THz emission at *B* = 0 T. a Excitation power dependence of sample transmittance at 800 nm. **b** The THz waveforms measured at

different excitation power. **c** Fourier transform spectra for THz waveforms at 5 different excitation power, and (**d**) the normalized spectra of (**c**).

Supplementary Note 5. Laser intensity dependence of thermoelectric current

In this session, we try to elaborate the dependence of thermoelectric current on laser intensity for photothermoelectric response. After laser pulse absorption, the photocarriers will be thermalized by rapid carrier-carrier scattering and reach a quasi-equilibrium Fermi-Dirac distribution that can be characterized by instantaneous electron temperature T_e. Due to the spatial temperature gradient of T_e, there is a thermoelectric current response, which can be written as $J_i = \alpha_{ij}\nabla T_{ej}$. In general, both the coefficient α_{ij} and the temperature gradient ∇T_{ej} is dependent on the electron temperature and they are discussed separately below.

Firstly, we consider the temperature gradient ∇T_e. Ignoring the thermal transfers to lattice and the environment during the first few picoseconds after the photoexcitation, the increase of instantaneous thermal energy of the electron system induced by laser absorption is

$$\int_{T_0}^{T_e} C_e(T_e)dT_e = \beta(z)I \quad (3)$$

where T_0 is the temperature before excitation, which is room temperature in our experiment; $C_e(T_e) = \gamma T_e$ is the electron specific heat, which is proportional to electron temperature; $\beta(z)$ is the relative light absorption at depth of z in Cd_3As_2 from the illuminated surface and I is the laser fluence. The instantaneous electron temperature can be written as:

$$T_e = \sqrt{\frac{2\beta(z)I}{\gamma} + T_0^2} \quad (4)$$

In our experiment, the magnitude of $\beta(z)$ should be about 10^5 cm^{-1}, γ ~ 70 μJ cm^{-3} K^{-2} [19], T_0 ~ 300 K, the average laser fluence I is in range of 5 ~ 90 μJ cm^{-2} in the laser intensity dependent measurements. T_e can be estimated to be from 40 K (5 μJ cm^{-2}) to 400 K (90 μJ cm^{-2}) above the room temperature. This estimation is consistent with Tr-ARPES measurement[20]. As the next step, the temperature gradient can be written as:

$$\nabla T_e = \frac{dT_e}{dz} = \frac{I}{\gamma\sqrt{\frac{2\beta(z)I}{\gamma} + T_0^2}}\frac{d\beta(z)}{dz} \quad (5)$$

For the in-plane Seebeck effect described in the maintext, the temperature gradient should follow the similar relation with I.

Then we consider the thermoelectric conductivity α_{ij}. Without magnetic field, α_{ij} can be written as $\alpha_{ii} = \sigma_{ii}S_{ii}$, where S_{ii} is the Seeback coefficient and σ_{ii} is the electrical conductivity. At weak magnetic field limit, both coefficients (S_{ii} and σ_{ii}) are nearly isotropic[21-23], and the value of S_{ii} and $\sigma_{ii} = \rho_{ii}^{-1}$ can be obtained from transport measurements in the literature[23]. S_{ii} show linear dependence on T_e: S_{ii} ~ 0.19 μV K^{-2} × T_e; ρ_{ii} is available in the temperature range from 10 K to 500 K in the literature[23]. Outside the temperature range, ρ_{ii} is calculated though linear extrapolation using the experimental data from 400 K to 500 K. With clear dependence of α_{ii} and ∇T_e on T_e, the dependence of J on the excitation intensity can be simulated numerically. The simulation results match experimentally measured excitation intensity dependence as shown in Fig. 2a of the maintext. In the meantime, this data can also be fitted by a power law: $E_{THz} \propto I^{0.66}$as shown in Supplementary Fig. 5.

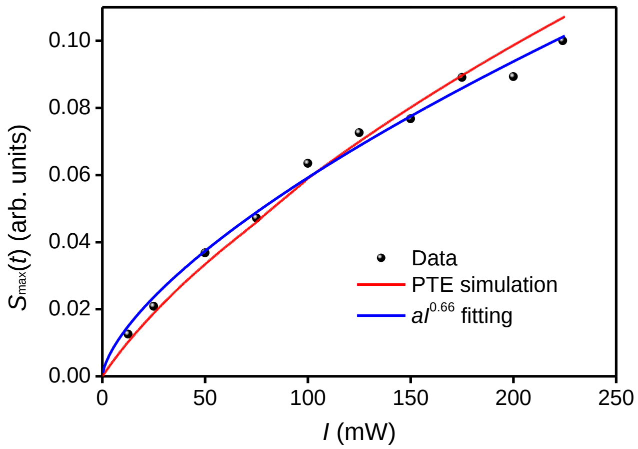

Supplementary Fig. 5 The laser intensity dependence of the peak field of emitted THz pulse. The red line is simulated power dependence according to the thermoelectric response calculation, and the blue line is a power law fitting.

We note the above simulation only concerns the thermoelectric response from electrons and the response from holes is not considered. This is because the transport and thermal properties of the holes in Cd_3As_2 are not available in the literatures so far. Considering the ambipolar transport nature during the first few picoseconds after the excitation, the contribution of holes to the THz emission cannot be ignore, but it may also follow similar power dependence with electrons according to the experimental results.

When a magnetic field is applied, the Nernst thermoelectric conductivity can be written as $\alpha_{ij} = \sigma_{ii}S_{ij} + \sigma_{ij}S_{jj}$. However, the temperature dependence of the coefficients related to Nernst effect are not available either, so that it is not feasible to simulate numerically. According to Supplementary Eq. 5, $I^{1/2}$ excitation intensity dependence obtained from experiment (as shown in Fig. 3a of maintext) requires the coefficient α_{ij} to have a sublinear dependence on T_e and tends to saturate at high temperature ($T_e^2 \gg T_0^2$, i.e., at high laser intensity).

Supplementary Note 6. THz waveform under opposite magnetic field

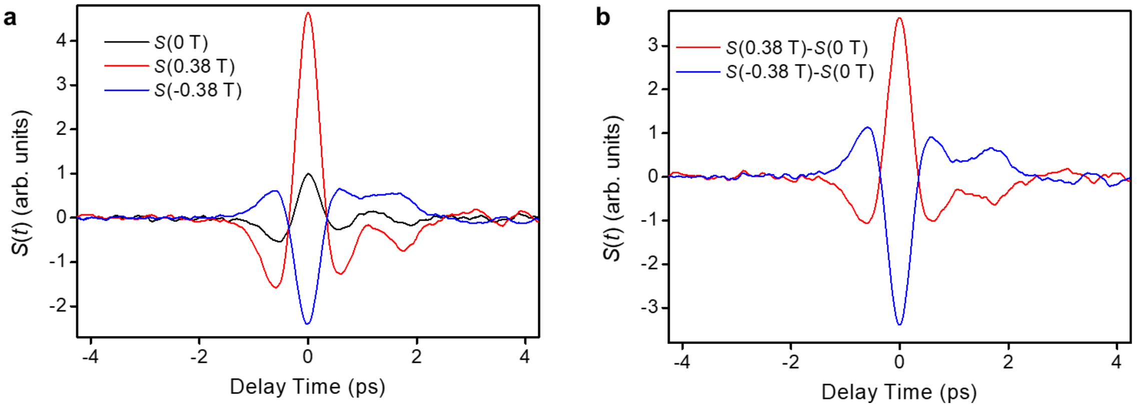

Supplementary Fig. 6 THz waveform under opposite magnetic field. a THz emissions from a 130 nm-Cd_3As_2/mica sample (which has a relatively large THz signal at 0 T) at B = 0 T and ±0.38 T. **b** The

THz waveforms at $B = \pm 0.38$ T with background signal at $B = 0$ T subtracted. The THz waveforms as result of magnetic field are exactly opposite to each other for $B = \pm 0.38$ T.

Supplementary Note 7. Magnetic field dependence of THz waveforms

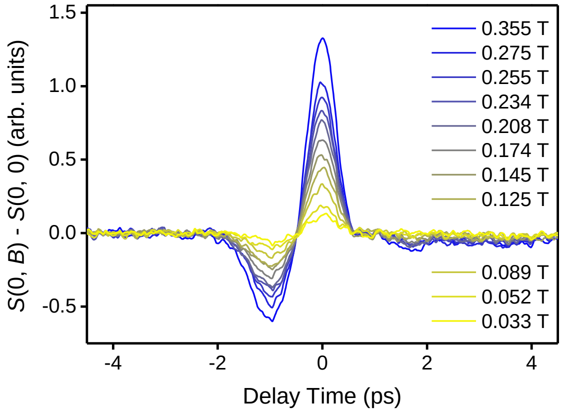

Supplementary Fig. 7 Magnetic field dependence of THz waveforms. THz waveforms measured at different in-plane magnetic field intensity with background signal at $B = 0$ T subtracted.

Supplementary Note 8. THz emission from Cd_3As_2 film

In this part, we provide experimental evidence that the THz emission is from the Cd_3As_2, not from the CdTe buffer layer and the substrate. We measured the THz emission with a 10 nm-CdTe/mica sample as shown in Supplementary Fig. 7. It shows very weak THz emission signal no matter with or without magnetic field, which are about 5 times smaller than the THz emission from the Cd_3As_2/CdTe/mica sample without magnetic field. Therefore, this comparison measurement indicates the THz emission is from Cd_3As_2 film, not from CdTe buffer layer or mica substrate.

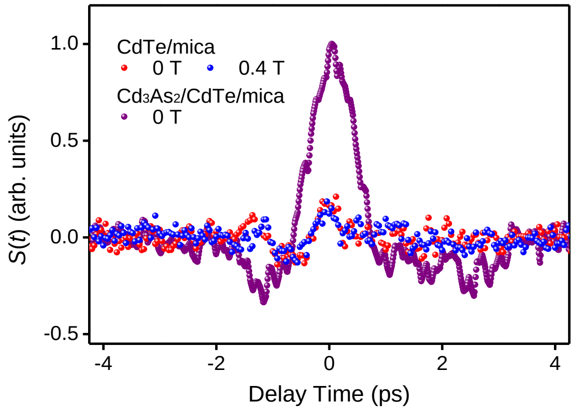

Supplementary Fig. 8 THz emissions from 10 nm-CdTe/mica at B = 0 T and 0.4 T and from Cd_3As_2/CdTe/mica at B = 0 T. The peak amplitude of THz emission from Cd_3As_2/CdTe/mica is set as 1 in the plot.

Supplementary References

1. Jia, Z. et al. Thermoelectric signature of the chiral anomaly in Cd_3As_2. *Nature Communications* **7**, 13013 (2016).

2. Liang, T. et al. Anomalous Nernst Effect in the Dirac Semimetal Cd_3As_2. *Physical Review Letters* **118**, 136601 (2017).

3. Wang, P. et al. Giant Nernst effect and field-enhanced transversal zNT in $ZrTe_5$. *Physical Review B* **103**, 045203 (2021).

4. Xu, X. et al. Thermoelectric transport and phonon drag in Weyl semimetal monochalcogenides. *Physical Review B* **104**, 115164 (2021).

5. Watzman, S. J. et al. Dirac dispersion generates unusually large Nernst effect in Weyl semimetals. *Physical Review B* **97**, 161404 (2018).

6. ZHOU, H., WANG, J., LOU, S., WANG, H. & ZHOU, S. Synthesis and thermoelectric properties of tungstentelluride nanobelts. *Scientia Sinica Physica, Mechanica & Astronomica* **49**, 047301 (2019).

7. Zhu, Z. et al. Quantum Oscillations, Thermoelectric Coefficients, and the Fermi Surface of Semimetallic WTe_2. *Physical Review Letters* **114**, 176601 (2015).

8. Wang, H., Schechtel, E., Pei, Y. & Snyder, G. J. High Thermoelectric Efficiency of n-type PbS. *Advanced Energy Materials* **3**, 488-495 (2013).

9. Zhao, L.-D. et al. Ultralow thermal conductivity and high thermoelectric figure of merit in SnSe crystals. *Nature* **508**, 373-377 (2014).

10. Liu, W. et al. Convergence of Conduction Bands as a Means of Enhancing Thermoelectric Performance of n-Type $Mg_2Si_{1-x}Sn_x$ Solid Solutions. *Physical Review Letters* **108**, 166601 (2012).

11. Christenson, E. L. Effects of Transition Metal Solutes on the Thermoelectric Power of Copper and Gold. *Journal of Applied Physics* **34**, 1485-1491 (1963).

12. Bel, R., Jin, H., Behnia, K., Flouquet, J. & Lejay, P. Thermoelectricity of URu_2S_2: Giant Nernst effect in the hidden-order state. *Physical Review B* **70**, 220501 (2004).

13. Wei, B. et al. Nernst effect of the intermediate valence compound $YbAl_3$: revisiting the thermoelectric properties. *Journal of Physics: Condensed Matter* **27**, 105601 (2015).

14. Lin, X., Zhu, Z., Fauqué, B. & Behnia, K. Fermi Surface of the Most Dilute Superconductor. *Physical Review X* **3**, 021002 (2013).

15. Heremans, J. P., Thrush, C. M. & Morelli, D. T. Thermopower enhancement in lead telluride nanostructures. *Physical Review B* **70**, 115334 (2004).

16. Meinero, M. et al. Unusual thermoelectric properties of $BaFe_2As_2$ in high magnetic fields. *Physical*

Review B **98**, 155116 (2018).

17. Kolincio, K. K. et al. Giant Nernst effect in the incommensurate charge density wave state of P4W12O44. *Physical Review B* **94**, 241118 (2016).

18. Planken, P. C. M., Nienhuys, H.-K., Bakker, H. J. & Wenckebach, T. Measurement and calculation of the orientation dependence of terahertz pulse detection in ZnTe. *J Opt Soc Am B* **18**, 313-317 (2001).

19. Wu, D. et al. Large and anisotropic linear magnetoresistance in bulk stoichiometric Cd_3As_2 crystals. *Science China Physics, Mechanics & Astronomy* **58**, 1-6 (2015).

20. Bao, C. et al. Population inversion and Dirac fermion cooling in 3D Dirac semimetal Cd_3As_2. Preprint at https://arxiv.org/abs/2112.09371v1 (2021).

21. Wang, H. et al. Magnetic-field enhanced high-thermoelectric performance in topological Dirac semimetal Cd3As2 crystal. *Science Bulletin* **63**, 411-418 (2018).

22. Xiang, J. et al. Large transverse thermoelectric figure of merit in a topological Dirac semimetal. *Science China Physics, Mechanics & Astronomy* **63**, 237011 (2019).

23. Wang, H. et al. Magnetic Field-Enhanced Thermoelectric Performance in Dirac Semimetal Cd_3As_2 Crystals with Different Carrier Concentrations. *Advanced Functional Materials* **29**, 1902437 (2019).
